# Next-generation sequencing of flow-sorted wheat chromosome 5D reveals lineage-specific translocations and widespread gene duplications

**DOI:** 10.1186/1471-2164-15-1080

**Published:** 2014-12-09

**Authors:** Stuart J Lucas, Bala Anı Akpınar, Hana Šimková, Marie Kubaláková, Jaroslav Doležel, Hikmet Budak

**Affiliations:** Faculty of Engineering and Natural Sciences, Sabanci University, Orhanlı, 34956 Tuzla, Istanbul, Turkey; Sabanci University Nanotechnology Research and Application Centre (SUNUM), Sabanci University, Orhanlı, 34956 Tuzla, Istanbul, Turkey; Centre of the Region Haná for Biotechnological and Agricultural Research, Institute of Experimental Botany, Sokolovská 6, CZ-77200 Olomouc, Czech Republic

**Keywords:** Wheat genome, Chromosome sorting, *Triticum aestivum*, Genome zipper, Triticeae genome, Chromosome arm shotgun, Comparative grass genomics

## Abstract

**Background:**

The ~17 Gb hexaploid bread wheat genome is a high priority and a major technical challenge for genomic studies. In particular, the D sub-genome is relatively lacking in genetic diversity, making it both difficult to map genetically, and a target for introgression of agriculturally useful traits. Elucidating its sequence and structure will therefore facilitate wheat breeding and crop improvement.

**Results:**

We generated shotgun sequences from each arm of flow-sorted *Triticum aestivum* chromosome 5D using 454 FLX Titanium technology, giving 1.34× and 1.61× coverage of the short (5DS) and long (5DL) arms of the chromosome respectively. By a combination of sequence similarity and assembly-based methods, ~74% of the sequence reads were classified as repetitive elements, and coding sequence models of 1314 (5DS) and 2975 (5DL) genes were generated. The order of conserved genes in syntenic regions of previously sequenced grass genomes were integrated with physical and genetic map positions of 518 wheat markers to establish a virtual gene order for chromosome 5D.

**Conclusions:**

The virtual gene order revealed a large-scale chromosomal rearrangement in the peri-centromeric region of 5DL, and a concentration of non-syntenic genes in the telomeric region of 5DS. Although our data support the large-scale conservation of Triticeae chromosome structure, they also suggest that some regions are evolving rapidly through frequent gene duplications and translocations.

**Sequence accessions:**

EBI European Nucleotide Archive, Study no. ERP002330

**Electronic supplementary material:**

The online version of this article (doi:10.1186/1471-2164-15-1080) contains supplementary material, which is available to authorized users.

## Background

Bread wheat (*Triticum aestivum* L.) is among the world’s most important crops, occupying 17% of all cultivated land and supplying about 55% of carbohydrates for human consumption [1]. However, its very large (~17 Gb), polyploid and repetitive genome presents major challenges to genome sequencing. *T. aestivum* is an allohexaploid derived from serial hybridization events between three different diploid wheat ancestors with A, B and D genomes [2], resulting in each of its 7 chromosomes being present in 3 phylogenetically related but divergent sub-genomes (2n = 6× = 42, genome formula AABBDD). Therefore, many genetic features have 3 homoeologous variants, some of which may not be functional. Over the last few years, through the development of high-throughput next generation sequencing (NGS) technologies, the sequencing of very large genomes has become increasingly feasible; however, the assembly of these sequence reads particularly from the highly repetitive regions remains challenging. In the case of bread wheat, the International Wheat Genome Sequencing Consortium (IWGSC) has developed a roadmap for sequencing projects [3], using flow sorting techniques to isolate individual chromosomes and chromosome arms. This strategy has been used to produce chromosome-specific BAC (bacterial artificial chromosome) libraries for genomic studies [4, 5], such as BAC-end sequencing (BES) of chromosome 3B and the long arm of chromosome 1A, which provided initial samples of 1.1% (3B) and 1.43% (1AL) of the sequences of these chromosomes [6, 7]. Following these studies, shotgun sequencing at relatively low coverage has been used to generate comprehensive surveys of large grass genomes, including in-depth exploration of gene content and organization. This approach was first demonstrated by Mayer *et al.*[8], who characterized barley chromosome 1H, tagging 5,126 genes and, by exploiting synteny with rice and sorghum sequences, ordering 1,987 of these into a virtual gene map. This was subsequently extended to the whole barley genome [9]; in the meantime, similar sequence surveys have been reported for *T. aestivum* chromosomes 4A , 5A, 6B & 7BS, and a comparison of the homoeologous group 1 chromosomes from both species [10–14]. Each of these studies has enabled the eludication of the structure of the respective chromosome in much greater detail than it was previously possible. In addition, utilizing 454 sequencing technology, the entire *T. aestivum* genome has been sequenced to a 5× coverage; from which several low copy number sequences and gene models based on orthologous sequences from other grass species were proposed [15]. Very recently, the IWGSC released Illumina survey sequences and predicted gene models from each individually flow-sorted chromosome arm [16]. While these studies have considerably contributed to our understanding of the wheat genome on a global scale and the draft sequences generated so far greatly benefit the wheat research community, the ultimate goal of a complete and reference-quality genome sequence still requires much additional work. To date, only chromosome 3B has been sequenced to this standard, using a BAC-by-BAC approach [17]; efforts are ongoing to extend our knowledge of the rest of the wheat genome.

The modern bread wheat genome is thought to have arisen from the hybridization of the D sub-genome with the ancestral tetraploid wheat during the establishment of modern agriculture, possibly as recently as ~10,000 years ago [2]. Phylogenetic analysis also places the divergence of the D-genome from its nearest diploid relative (*Aegilops tauschii)* more recently than that of the A or B genomes [18]. As relatively few *Ae. tauschii* genotypes were involved in the origin of *T. aestivum*[19], the D-genome is typically lacking in genetic diversity and polymorphism, resulting in fewer loci on available genetic maps [20], although the D genome is also therefore a natural target for introgressing desirable traits from *Ae. tauschii.* Chromosome 5D, at 748 MB in size [5] comprises 15.1% of the D sub-genome and 4.4% of the entire bread wheat genome. This chromosome harbours genes for a number of agriculturally important traits; for instance, grain hardness, the single most important determinant of wheat end-use quality, is controlled by two puroindoline proteins that are expressed from the *Pina-D1* and *Pinb-D1* loci located close to the short arm telomere of 5D [21]. Vernalization and flowering time habits, which are vital for the adaptation of wheat to different climates, depend on at least 4 *Vrn* loci, 2 of which have homoeologs on chromosome 5D; mutations in both *Vrn-D1* and *Vrn-D4* have been shown to influence vernalization [22, 23]. There are also several disease response loci that have been mapped to 5D, of which only the leaf rust resistance gene *Lr1* has been cloned so far [24].

In this study, we carried out shotgun sequencing of flow-sorted bread wheat chromosome 5D using 454 technology, thereby providing a complementary dataset to the previously published whole genome shotgun [15] and chromosome-by-chromosome Illumina survey sequences [16]. We assembled a catalogue of genes present on 5D and exploited synteny with other grass genomes to place 2138 into a virtual gene order, enabling us to assess the consistency of NGS sequence surveys and suggest refinements to the existing data. We also highlight structural and evolutionary features of chromosome 5D, including gene duplications and translocations and large-scale chromosome rearrangements. These observations will be valuable for cloning of important trait genes and the future mapping and reference-quality sequencing of chromosome 5D.

## Results

### Isolation and survey sequencing of wheat chromosome 5D

The short and long arms of chromosome 5D (hereafter 5DS and 5DL, respectively) were isolated from double ditelosomic lines (see Methods, Additional file 1). The purity of the DNA was estimated to be 90.18% for 5DS and 85.5% for 5DL, the impurities consisting of fragments from other chromosomes. As the isolation of sufficient DNA for direct sequencing is prohibitively resource and time-consuming, the sorted telosomes were amplified by Multiple Displacement Amplification (MDA) giving yields of 15.81 μg (5DS) and 9.64 μg (5DL). These DNA were fragmented and each telosome was sequenced using Roche/454 Titanium technology to give 1.34 – 1.61 fold coverage (Table 1).

**Table 1:** **Summary of sequencing statistics for 5D chromosome arms**

Arm	Size ^1^ (Mbp) S	No. of reads N	Mean read length L (bp)	Total read length (Mbp)	Coverage ^1^	Purity	Representation probability ^2^
**5DL**	490	2,271,366	347.25	791	1.61x	85.5%	0.684
**5DS**	258	937,264	370.28	347	1.34x	90.2%	0.667

^1^Calculated based on cytogenetic estimate of chromosome arm length [5].

^2^Calculated as: P = [1 – (1 - L/S)^N^ ] x Purity.

### Sequence assembly and detection of repetitive elements

Previous studies have suggested that bread wheat chromosomes consist of 80-90% repetitive elements, the majority of which belong to the Long Terminal Repeat (LTR) class of retrotransposons [6, 7]. LTR elements can be several kilobases long, making accurate *de novo* sequence assembly impossible from reads of 300–400 nt, particularly at low chromosome coverage. Therefore, a combination of assembly and sequence similarity methods were utilized to identify transposable elements (TEs) and other repetitive sequences (Table 2, Additional files 2 &3). A summary of all repeat families identified is given in Additional file 4. After eliminating all detected repetitive elements, the remaining non-repetitive sequence reads had a total length of 84.6 Mb for 5DS and 201 Mb for 5DL; these were used for all the following sequence comparisons and gene searches.

**Table 2:** **Summary of assembly statistics and filtering of survey sequences**

Chromosome arm	5DS	5DL
**Aligned reads no.**	578,521	1,282,886
**Aligned reads %**	61.72	56.48
**Aligned bases no.**	190,520,827	385,146,579
**Aligned bases %**	54.90	48.78
**Singleton reads**	256,024	672,427
**Contigs (>100 bases)**	47,674	95,485
**Bases in contigs**	35,171,389	67,393,211
**Non-aligned bases**	156,537,017	404,467,896
**Estimated arm length (Contigs + non-aligned)**	191.7 MB	471.9 MB
**Deep contig reads no.**	198,518	438,597
**Deep contig reads %**	21.18	19.31

### Discovery of orthologous gene sequences on chromosome 5D

**Figure 1:**
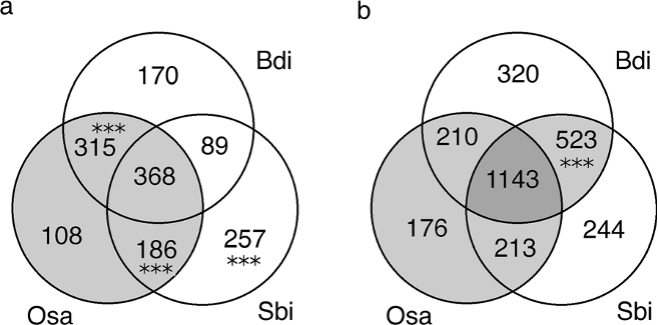
**Gene conservation between sequenced grass genomes and 5D.** Venn diagram showing the number of different genes from the genomes of *B. distachyon* (Bdi), *O. sativa* (Osa) and *S. bicolor* (Sbi) with homologous sequences on 5DS (**a**.) and 5DL (**b**.). Stars highlight homologs matching each species that form a significantly higher proportion of conserved orthologous genes on one chromosome arm than the other (***p < 0.001, Fisher’s exact test). Light grey shading highlights groups of conserved orthologs for which more than 70% also found an homolog in UniGene/UniProt; dark grey shading, more than 90%.

The masked and filtered sequence reads produced above were examined for potential gene coding sequences by similarity searches against all predicted proteins from the sequenced grass genomes (*Brachypodium distachyon, Oryza sativa* and *Sorghum bicolor*), a subset of UniProt containing only monocotyledon proteins, and UniGenes derived from all available *T. aestivum, Hordeum vulgare, Panicum virgatum, Saccharum officinarum,* and *Zea mays* ESTs (described in Methods). After removing low quality alignments and amplification artifacts, the hits to each dataset were combined, and 53,163 reads from 5DL and 26,535 from 5DS were found to match a gene coding sequence from at least one of the databases (Table 3). Notably, 18,771 (70.7%) and 33,619 (59.9%) of the reads from 5DS and 5DL, respectively, yielded a match in only one of the databases; whereas only 1210 (4.5%) of reads from 5DS and 4208 (7.5%) of reads from 5DL matched all 3 sequenced grass genomes. These observations suggest that 5DL contains a higher proportion of conserved genes than 5DS, which shows greater divergence from other grass species in terms of orthologous genes. These sequences may include contaminating fragments from other chromosomes, however, the total length of such fragments would give only a 0.001× coverage of the rest of the genome, making it highly unlikely that any contaminating gene sequence would be represented by more than one unique sequence read. Therefore, to avoid contaminants, gene sequences which were only matched by a single sequence read were eliminated. Additionally, a small number of UniGene and UniProt sequences that matched >50 reads were excluded as probable repetitive sequences. After applying these quality criteria, the number of unique genes from *B. distachyon, O. sativa* and *S. bicolor* with matches to the survey sequences was evaluated (Figure 1). A total of 1493 genes were found with homologous sequences on 5DS, and 2829 on 5DL. Interestingly, 5DS contained a statistically higher proportion of sequences conserved with *Brachypodium* and rice but not sorghum, and of genes that matched sorghum but not *Brachypodium* (Figure 1a). In contrast, 5DL had a much higher representation of genes that matched *Brachypodium* and sorghum, but not rice (Figure 1b). Matches to the UniGene and/or UniProt datasets used provide evidence that a putative orthologous gene sequence is also present in other crop species that have not yet been fully sequenced. It was noted that while only 59% of putative conserved genes on 5DS were supported by UniGene and/or UniProt hits, 79% of those on 5DL were. In particular, of putative 5DL genes that were conserved among all 3 grass genomes, more than 90% had UniGene/UniProt matches, a significantly higher proportion than 5DS (Figure 1b; p < 0.001, Fisher’s exact test). Taken together, these results suggest that mutations accumulated within conserved gene sequences may not be distributed uniformly across chromosome 5D, and that the primary sequence of such genes on 5DL has been conserved more than those on 5DS. In addition, 2 or more sequence reads matched each of 4500 (5DL) and 1812 (5DS) UniGene/UniProt entries, but no predicted protein sequences from *Brachypodium*, sorghum and rice at the same stringency (≥75% amino acid similarity over at least 30 amino acids). The great majority of these UniGene/UniProt entries were from *T. aestivum* and *H. vulgare,* suggesting that these represent gene sequences that have accumulated a large number of mutations during Triticeae evolution. These may include both genes with novel functions, and gene fragments/pseudogenes, an issue explored further below (“5D gene modelling and annotation”). These sequences are hereafter referred to as ‘non-conserved gene-like sequences’; full details of these and all the putative orthologous genes sampled are given in Additional file 5.

**Table 3:** **5D survey sequence reads with homology to 5 different gene datasets**

	Matching reads from 5DS					Matching reads from 5DL				
	*Bdi*	*Osa*	*Sbi*	UniG	UniP	*Bdi*	*Osa*	*Sbi*	UniG	UniP
**Total read no.**	**5665**	**4035**	**4260**	**18521**	**8063**	**13413**	**9863**	**11054**	**39266**	**18844**
***B. distachyon***	* 1303*	2737	1826	2628	2265	* 5598*	6190	6852	7111	7117
***O. sativa***		* 374*	1618	2195	1975		* 4840*	5704	5767	6091
***S. bicolor***			* 861*	2216	1905			* 4988*	6364	6788
**Unigene set**				*13004*	3610				* 12055*	10660
**Uniprot set**					* 3229*					* 6138*
***Bdi + Osa + Sbi***	1210					4208				

Values given are the number of 454 reads matching the datasets indicated by row and column headings. Values in italics are the number of reads matching only a single dataset.

### tRNA repertoire of chromosome 5D

**Figure 2:**
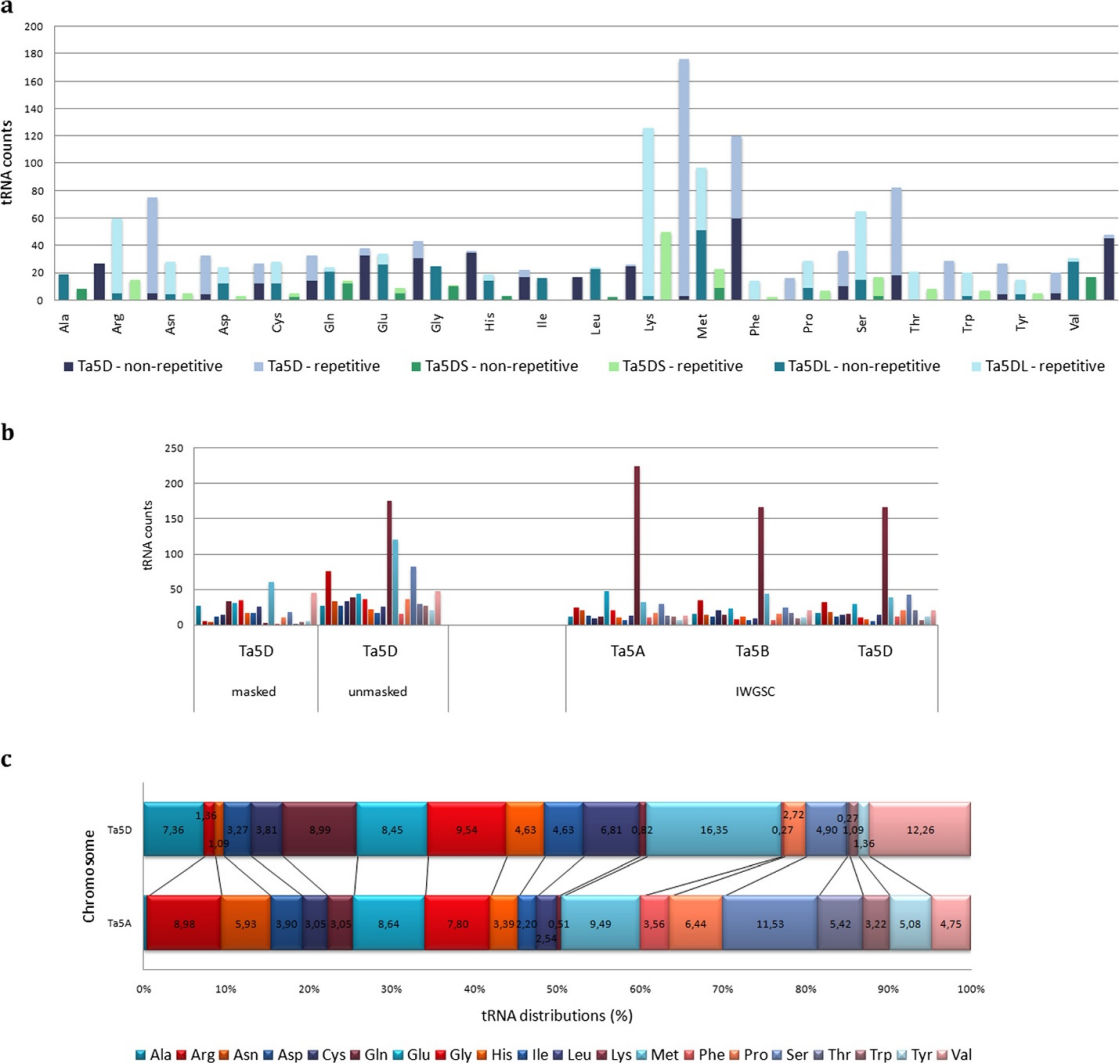
**Putative tRNA genes predicted from sequence reads.** Putative tRNA gene predictions for **a**. repetitive and non-repetitive 5D sequence reads, **b**. 5D sequence reads compared to predictions from IWGSC Illumina contigs of homeologous group 5 chromosomes [16], **c**. Non-repetitive 5D sequence reads compared to non-repetitive 5A sequences obtained by the same sequencing technology [10].

A recent study revealed an unusual abundance for putative tRNA^Lys^ genes among repeat-containing sequences from wheat chromosome 6B [14]. Sequence reads (both repeat-masked and unmasked) from both arms were screened for the presence of putative tRNA gene sequences, and, similarly, an overabundance of putative tRNA^Lys^ species was predicted from unmasked sequence reads, while masked reads did not yield such a dramatic bias in any tRNA species (Figure 2a). This implies that either some repetitive sequences resemble tRNA gene sequences, particularly for tRNA^Lys^, or that some genuine tRNA genes are located inside repetitive regions of the chromosome. The tRNA gene content of the Illumina sequence contigs from *T. aestivum* group 5 chromosomes also exhibited the same pattern for all homeologous group 5 chromosomes, indicating that the abundance of tRNA^Lys^ species is a shared phenomenon and is not an artefact caused by different sequencing technologies. Consistent with these observations and the conclusions of the previous report [14], it is likely that some tRNA genes have been captured by TEs and co-expanded as the TEs proliferated. This would be consistent with a genome-wide expansion of TEs containing tRNA^Lys^ after the formation of hexaploid wheat, as chromosomes from different homoeologous groups and ancient origins are equally affected (group 6 and 5 chromosomes are thought to originate from different ancestral chromosomes (A2 and A3/A12/A9, respectively, of an n = 12 ancestor) [25]. As the TE-driven expansion of tRNA genes located inside the repetitive portion of the genome suggests that many of these putative genes may be non-functional copies, the comparative tRNA content of non-repetitive sequences from different chromosomes were inspected for any functional differences.

In order to eliminate the differences in tRNA gene predictions resulting from the different sequencing and bioinformatics platforms used, previously published 454 survey sequences from 5A chromosome [10] were repeat-masked and screened for putative tRNA genes using the same procedure as 5D. The predicted tRNA gene density on 5A was almost 1.5 times greater than 5D (0.71 vs. 0.49 tRNA genes/Mb, respectively). The two homeologous chromosomes not only differ in total tRNA gene content, but also different tRNA genes were enriched on 5A and 5D (Figure 2c), which may have implications for the translational machinery.

### Syntenic relationships between 5D and other grasses

**Figure 3:**
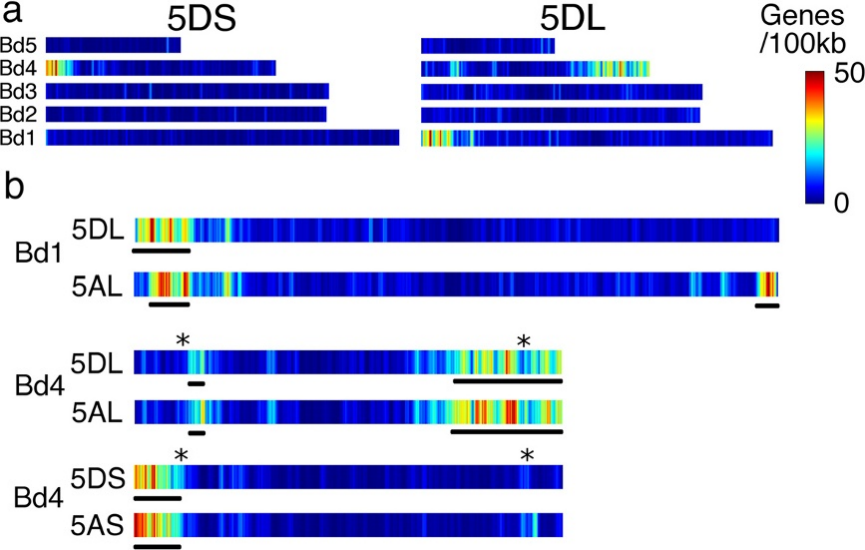
**Distribution of 5D genes conserved with other grass genomes.** All heat maps were drawn using a sliding window approach, with a window size of 500 kb and a step size of 50 kb. **a**. Heat map showing distribution of 5D sequence reads with homology to genes on *B. distachyon* (Bd) chromosomes. **b**. Heat maps comparing the distribution of conserved sequences from chromosomes 5D and 5A on the syntenic *B. distachyon* chromosomes Bd1 and Bd4. Black bars under each heat map highlight the major syntenic blocks, while * shows the 2 regions of Bd4 which contained a mixture of sequences conserved with 5DS and 5DL.

**Figure 4:**
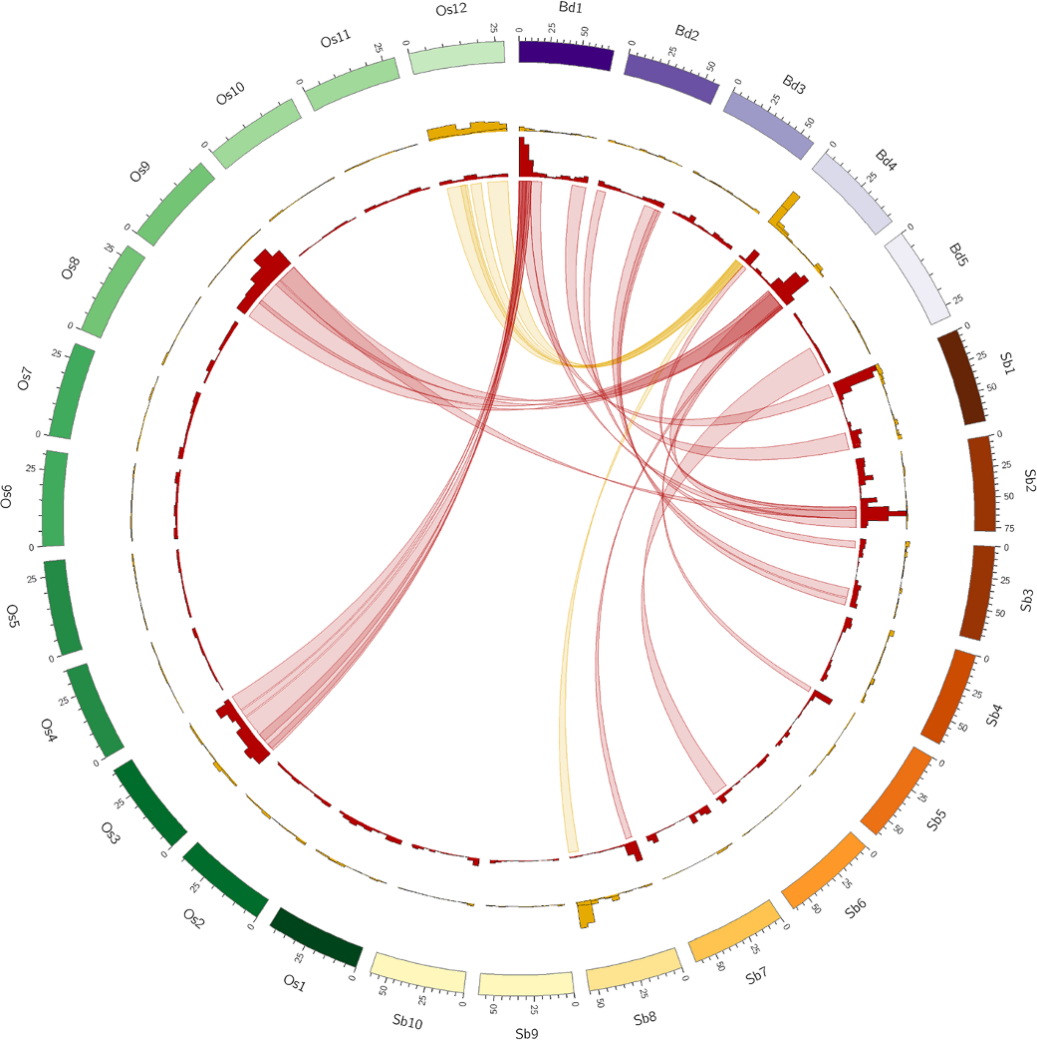
**Syntenic relationships between 5D and sequenced grass species.** Circle plot in which reads from 5DS and 5DL are grouped into ribbons linking the chromosomes with which they show homology. Chromosomes of *B. distachyon* (Bd)*, S. bicolor* (Sb) &*O. sativa* (Os) are shown as coloured bars around the outside of the circle. The relative abundance of syntenic reads by the position along each chromosome segment is shown by the histograms; yellow indicates genes matching 5DS, red indicates genes matching 5DL. Blocks containing 50 or more genes in 1 Mb that are conserved in 2 or more sequenced species are joined by ribbons, yellow for 5DS and red for 5DL.

**Figure 5:**
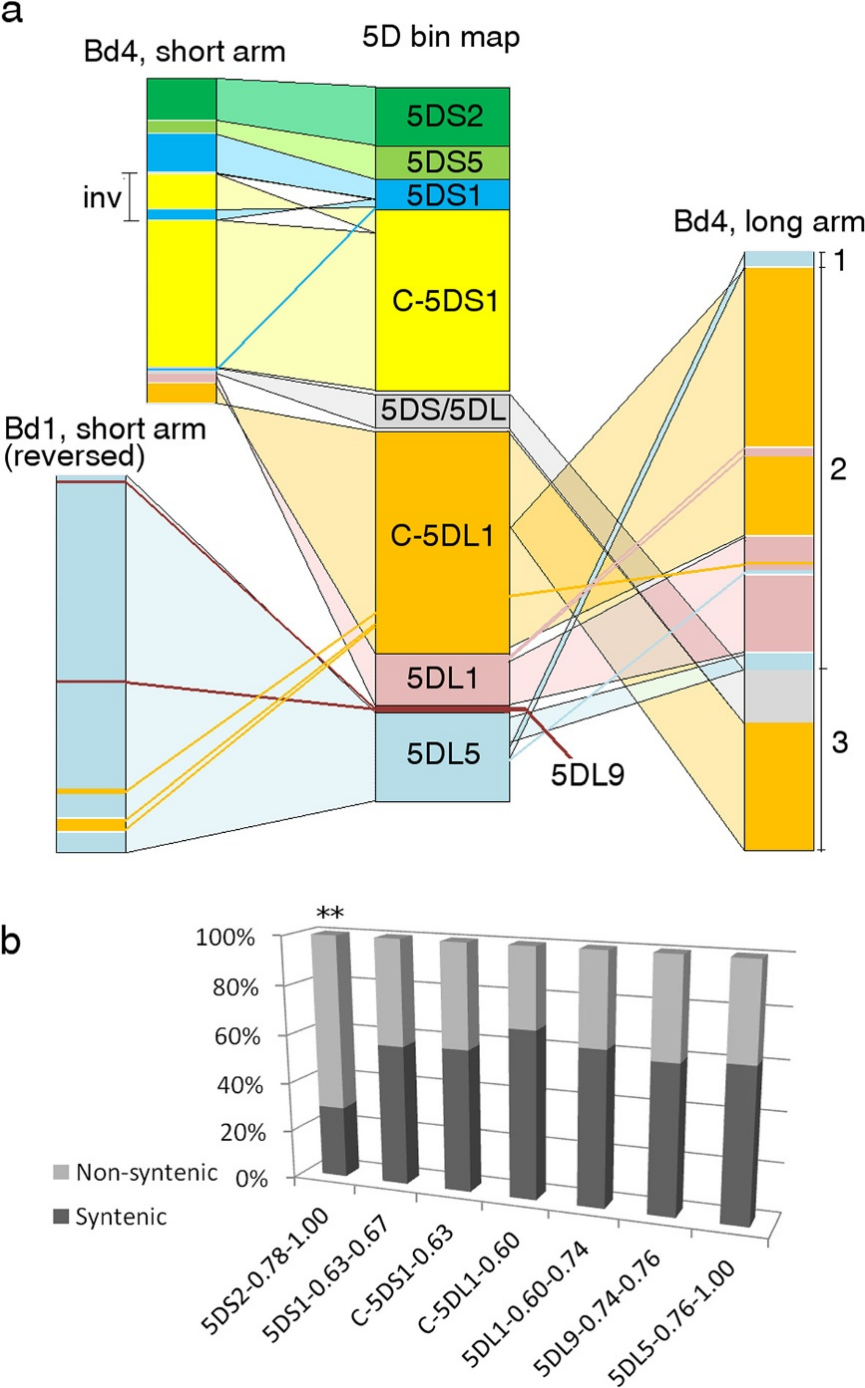
**Chromosome structure comparison between** ***T. aestivum*** **5D and** ***B. distachyon.*** **a**. Cartoon showing co-linearity between 5D deletion bins and *B. distachyon* chromosomes 1 (Bd1) and 4 (Bd4), and major rearrangements. Pale coloured bands show large regions of co-linear genes. Coloured lines show smaller translocations where one or a few genes were mapped to a different deletion bin. inv: probable inversion in 5DS relative to Bd4. 1,2,3: segments of 5DL that are rearranged relative to Bd4. **b**. Graph showing relative contribution of syntenic and non-syntenic genes to each deletion bin, for all genes that were also mapped to a deletion bin. Asterisks indicate statistically significant differences in the composition of bin 5DS2, calculated using Fisher’s exact test (**p < 0.01). Bin 5DS5 was omitted because too few genes mapped to this bin to draw meaningful conclusions.

The chromosomal locations of all genes from the *Brachypodium*, sorghum and rice genomes with homologues on 5D were used to identify conserved regions of these genomes with each chromosome arm (Figure 3 & Additional file 6), while matches to 2 or more genomes were used to highlight syntenic relationships between the model grasses (Figure 4). 5DS orthologous reads revealed a clear syntenic block, corresponding to the proximal end of *B. distachyon* chromosome 4 (Bd4), the distal end of *S. bicolor* chromosome 8 (Sb08), and *O. sativa* chromosome 12 (Os12) (Figures 3a,4). Immediately adjacent to this block in Bd4, another small segment showed high conservation with 5DL; however, genes from this region corresponded to Sb02 and Os09 (Figure 4). 5DL also contained a much larger syntenic block on the long arm of Bd4 that was also syntenic with Sb02 and Os09, and a second major syntenic block containing orthologous genes from the proximal ends of Bd1 and Sb01, which were also dispersed along Os03. The previously published survey sequences of chromosome 5A [10] obtained with the same sequencing technology were also re-analyzed using the same criteria applied to the 5D sequences, and the locations of conserved sequences from both chromosomes on syntenic regions of *B. distachyon* compared (Figure 3b). As expected, the regions of 5AL syntenic with Bd1 differed from 5DL in that part of the block at the proximal end of Bd1 was not present, while a new block was present at the distal end of the chromosome. This difference in synteny reflects the previously documented 4AL/5AL translocation [13, 26] and no other large scale differences in syntenic blocks were observed. However, there was evidence of fine structure variations within these blocks, which was also displayed in the relationships with the rice and sorghum genomes; for example, a significant minority of the 5DL sequences matching Bd4 mapped to Sb05, not Sb02 (Figure 4). Also, a large number of conserved genes on both chromosome arms fell outside these syntenic regions. A virtual gene order of wheat chromosome 5D was constructed using the ‘genome zipper’ approach [8]. First of all, co-linear genes were ordered according to the syntenic regions in *B. distachyon.* Then, 518 deletion bin-mapped wheat EST and SSR sequences were mapped onto the syntenic gene reads, some of which also had positions on the International Triticeae Mapping Initiative wheat reference genetic map. The resulting gene order and all mapped markers are given in Additional file 7. Based on these comparisons, 5DS was found to be largely co-linear with a section of Bd4 from Bradi4g00200 – Bradi4g07997, apart from a probable inversion including the genes from Bradi4g02840 – Bradi4g03750 (Figure 5a). The telomeric region of 5DL, corresponding to deletion bin 5DL5, was predominantly co-linear with the short arm of Bd1 in reverse order (Bradi1g15730 – Bradi1g00227), although a few genes from this region mapped to other deletion bins, giving evidence for several small-scale translocations within this region. The rest of 5DL was syntenic with most of the long arm of Bd4 (Bradi4g23910 – Bradi4g45397 though extensive rearrangements were also evident. A model that explains the observed data is presented in Figure 5a; if the region is divided into 3 segments, the segments starting from the centromeric region are present in the order 1-2-3 in Bd4, but in the reverse order 3-2-1 in 5DL. Within each segment co-linearity is largely maintained apart from a few small-scale translocations from segment 2 to other parts of the chromosome arm (region 1 may or may not be inverted). The boundary between segment 2 and segment 3 on Bd4 lies between Bradi4g38980 and Bradi4g39020; more genetically mapped markers are required to determine the precise boundary between segments 1 & 2, and any smaller rearrangements that might have occurred within each deletion bin. The centromeric region of 5DL also contains syntenic sequences from the short arm of Bd4 (Bradi4g08180 – Bradi4g08900), the segment adjacent to the region of Bd4 that is syntenic with 5DS (Figure 3b).

Interestingly, 2 small regions from Bd4, Bradi4g08020 – Bradi4g08140 and Bradi4g39020 – Bradi4g40770, were found to contain multiple small groups of genes that were conserved alternately with 5DS and 5DL (highlighted with stars in Figure 3b). These did not appear to be the result of contaminating DNA from the opposite arm in the isolated chromosomes, because very few genes were detected in both chromosome arms; also, several EST markers bin-mapped to 5DS were found in the latter region, which was expected to be co-linear to 5DL (see Additional file 7). Each of these heavily rearranged segments occurs at a putative breakpoint for a large-scale chromosomal translocation, and is inferred to contribute sequences close to the centromere of 5D.

In addition, the number of *B. distachyon* orthologous genes mapped to each 5D deletion bin was totalled (Figure 5b). Across the chromosome approximately 60% of bin-mapped genes were from the syntenic regions of Bd1 and Bd4, while 40% came from other chromosomes. The exception was the telomeric region of 5DS (deletion bin 5DS2) where only 29.2% of the mapped genes were syntenic. This was significantly lower than the rest of the chromosome (p = 0.0018, Fisher’s exact test), suggesting that this part of the chromosome contains a much higher proportion of non-syntenic genes.

**Figure 6:**
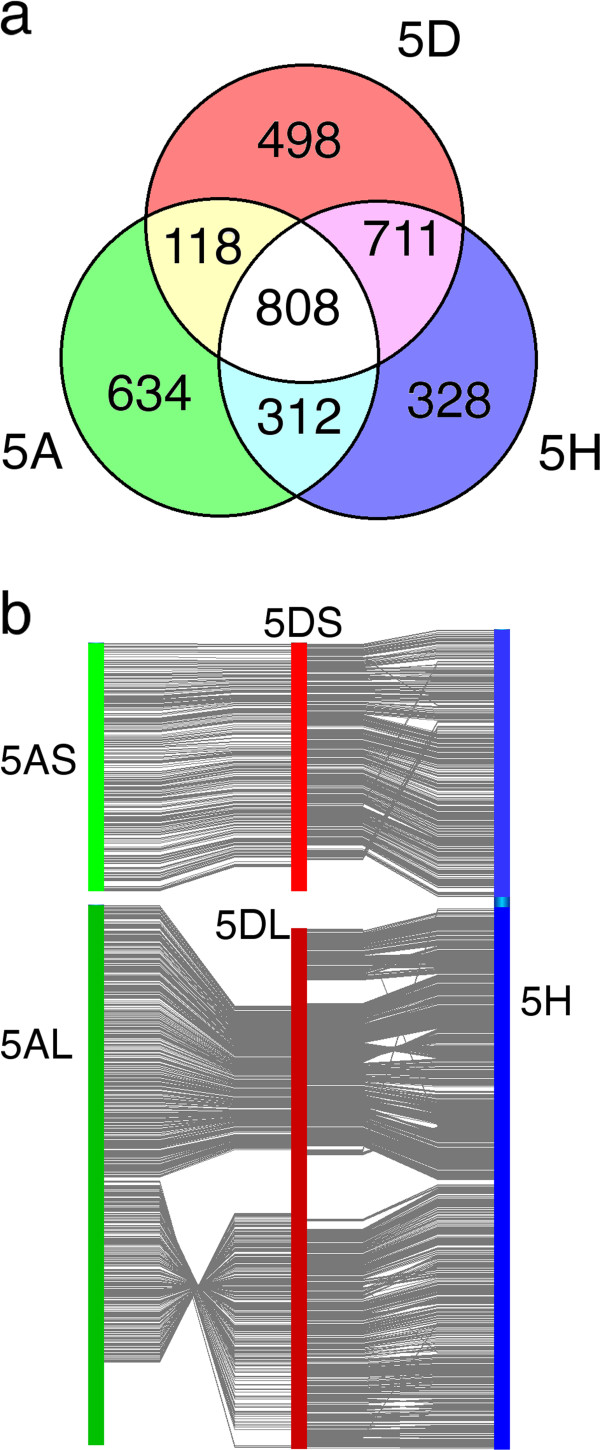
**Comparison of** ***T. aestivum*** **5D genome zippers with those of** ***T. aestivum*** **5A and** ***H. vulgare*** **5H.** The sequence of genes syntenic with *B. distachyon* on each of the three chromosomes was compared. **a**. Venn diagram showing the number of syntenic genes unique to and common to each pair of chromosomes. **b**. Comparison of the virtual gene order of 5D (centre) with 5A (left) and 5H (right). Each link shows the relative positions of a syntenic gene common to both chromosomes.

In order to assess the divergence between different Triticeae genomes, the virtual gene order of genes syntenic with *B. distachyon* identified on each arm of 5D was compared with those previously published for *T. aestivum* chromosome 5A [10] and *H. vulgare* chromosome 5H [9] (Figure 6). Only 37% of all the syntenic genes found on 5D (808/2135) were common to all three chromosomes, while 5D & 5H had more genes in common with each other than either of them did with chromosome 5A (Figure 6a). This is partly a result of the aforementioned 4AL/5AL translocation, but there is also a section syntenic with the long arm of *B. distachyon* chromosome 4 that is found near the centromere of 5DL and 5HL but was not found on 5AL (Figure 6b). Similarly a block from the middle of 5DL (corresponding to Bradi1g09140-Bradi1g15730) seems to be absent from both 5AL and 5H. As well as these large-scale variations, many differences involving isolated genes were found along the length of the chromosome. Throughout the syntenic regions, genes common to all three chromosomes were interspersed among those found on two or only one of them. In addition, several small-scale translocations were identified in the 5H gene order that have not been detected in 5A or 5D (Figure 6b). These differences may be due to the higher resolution genetic markers that were available to construct the virtual gene order of barley than wheat; as more sequence-based *T. aestivum* markers become available, some of these translocations may be found to be present on 5A and/or 5D as well.

In summary, these results suggest that syntenic regions form a highly conserved framework for the homeologous chromosomes; however, the subset of syntenic genes found within these regions may vary considerably even between closely related genomes. Moreover, the syntenic genes are interspersed with non-syntenic genes that show much greater variation.

### Wheat-specific genome rearrangements

Recently, the comparison of *Brachypodium*, rice and sorghum genes has been utilized to discover genes that might have been ‘moved’ or rearranged specifically in the genome of one of the three species [27]. Using a similar strategy, all 5D gene sequence reads conserved with all three model grasses but lying outside the 5D syntenic regions described above were analysed further for evidence of possible wheat-specific rearrangements. As above, genes matched by a single read were not considered; among the remaining non-syntenic genes, 86 (5DS) and 309 (5DL) were found to be conserved between 5D and all 3 grass genomes, corresponding to 294 & 905 sequence reads, respectively. Pseudogenes or gene fragments are less likely to be covered evenly by sequence reads than true genes, so, these non-syntenic genes were further examined as follows: genes covered by 4 or more sequence reads were visually examined for even coverage, while genes covered by only 2 or 3 sequence reads were divided into 2 or 3 equal length segments, and considered as genuine genes only if at least one read overlapped with each segment (see Additional file 8, as an example). Using these criteria, 32 and 129 non-syntenic, putatively ‘genuine’ genes were identified for 5DS and 5DL, respectively. Among these, non-syntenic genes that are syntenic between the other three grass genomes (*Brachypodium*, rice and sorghum) points to a probable wheat-specific genome rearrangement, which occurred after the Triticeae and the fully sequenced grasses diverged from their common ancestor. For instance, 19 sequence reads from 5DS matched Bradi1g17710, Os02t0167700 and Sb04g004540, homologous genes from regions which are syntenic with each other but not with *T. aestivum* 5D. Thus, these 19 sequences are presumed to represent a wheat homologue that was translocated to chromosome 5D, after wheat diverged from its common ancestor with *Brachypodium*. Twenty-two and 36 such genes from 5DS and 5DL respectively are proposed to have resulted from such wheat-specific rearrangements (Additional file 9). The possible functions of these genes, predicted from the respective *Brachypodium* protein sequence, include proteins related to transcriptional and translational machinery, along with several hypothetical proteins.

### 5D gene modelling and annotation

In order to model the coding sequences of genes identified here, and enable comparison with other *T. aestivum* gene datasets, the coding sequences of the genes homologous with 5D from *Brachypodium*, sorghum and rice were used as a reference on to which the non-repetitive 5D reads were assembled using gsMapper (Newbler 2.6). Multiple contigs mapping to different parts of the same reference were merged to give a single gene model, with gaps filled with runs of ‘n’. Similarly, UniGene and UniProt entries were used as a reference to assemble the non-conserved gene-like sequence reads. Previously published *T. aestivum* transcriptome sequences [15] were then mapped to all models, which were assigned a confidence value based on the proportion of bases mapped by transcriptome sequences. This yielded a total of 3147 High-Confidence gene models (60-100% transcript coverage), 2165 from 5DL and 982 from 5DS. A further 810 (5DL) and 332 (5DS) gene models, classed as ‘Low-Confidence’, had 20-60% transcript coverage. All remaining models were eliminated as probable gene fragments and pseudogenes. Generally speaking, a similar proportion of conserved genes and non-conserved gene-like sequences from both arms were supported by the transcriptome data, suggesting that many of the latter do represent functional genes. Interestingly, 5DS included relatively more gene models derived from non-conserved sequences than 5DL, especially among the Low Confidence models (p < 0.01, Fisher’s exact test). Assembly and transcriptome mapping statistics and sequences of all the High- and Low-Confidence gene models are given in Additional files 10 and 11.

**Figure 7:**
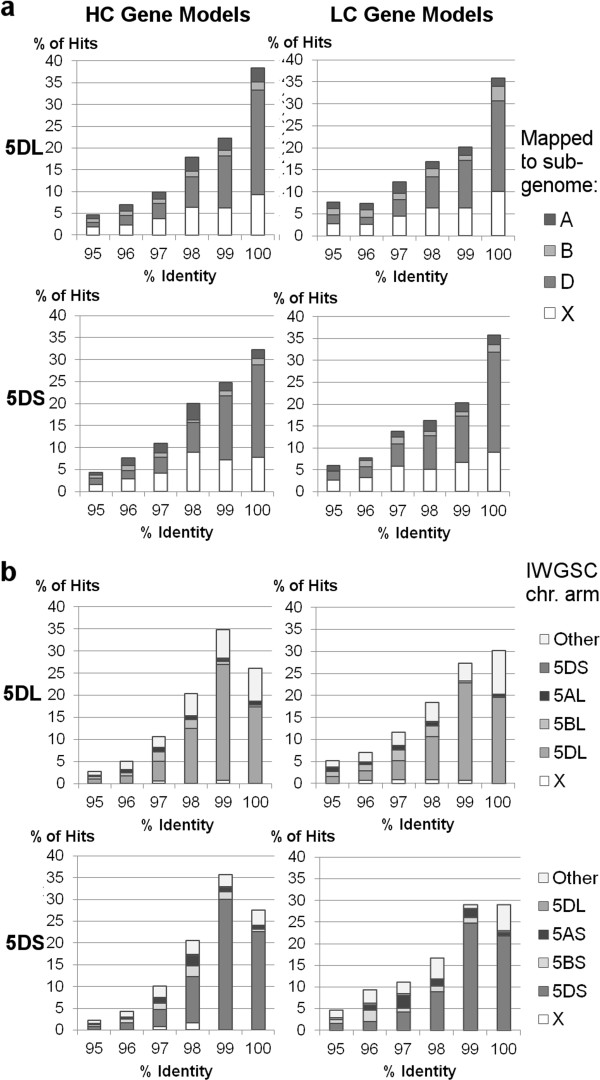
**Screening of gene models against other NGS datasets.** High- and low-confidence gene models from 5DS and 5DL were searched independently against gene models derived from **a**. whole genome shotgun 454 sequences [15] and **b**. chromosome-specific Illumina contigs [16]. Each bar of the histogram shows the % of all gene model hits for each comparison in a 1% sequence identity bin, starting from the value shown on the x-axis (e.g. ‘95%’ = 95.00-95.99%).

Two genome-wide NGS datasets have recently been released for *T. aestivum*, one from whole-genome shotgun 454 sequences [15] and the other from chromosome-specific Illumina sequences [16]. The gene models assembled from each are an extremely valuable resource for wheat researchers, although both groups state that these are draft sequences that will be improved by subsequent studies. The data presented in this paper represents a hybrid between the two, as it was generated from chromosome-specific 454 sequences. Therefore, we examined the consistency of the gene model sequences derived from all three studies by similarity searches, using a best reciprocal hit strategy with a minimum sequence identity of 95%. For all gene models that found a match, the sub-genomic/chromosomal locations to which their counterparts were assigned was compared, the distribution of which is shown in Figure 7. Of the gene models produced from our data, 2557 out of 2975 (5DL) and 1032 out of 1314 (5DS) matched a model from the whole genome shotgun sequences. The number of matches to Illumina sequences was slightly lower on 5DL (2319/2975) but markedly so on 5DS (853/1314). This difference could be partly explained by the fact that we used the same transcriptome data as the former study to confirm our gene models, but may also illustrate differences between the sequencing technologies and assembly pipelines used. The distribution of sequence identities was similar for both external datasets, although there was a higher proportion of 100% identical matches between our data and the previously reported 454 shotgun sequences, whereas more hits with the IWGSC data were found in the 99.00-99.99% identity range. These differences reflect the fact that Illumina sequencing was used for the latter study, and shows that 454 sequences are more likely to be consistent with each other, as each technology is prone to different kinds of sequence errors. The chromosome 5D gene models mostly matched models that were assigned to the D genome in the previous 454 study, although there were also a significant number that were unmapped but can now be assigned to 5D (Figure 7a). Similarly, in comparison with the IWGSC study, the majority of our gene models matched an Illumina gene model from the same chromosome arm, with 5DS slightly more consistent than 5DL, although some gave matches to other chromosomes even at 100% identity (Figure 7b). Also, for best hits with <98% identity, more than half were found on chromosomes other than 5D. These likely correspond to gene copies with slightly different sequences on other chromosomes; accordingly, it has previously been observed that homoeologs can be up to 99% identical with each other [28]. More best hits were found on 5B (~8% for both arms) than 5A (4% for 5DL, 7% for 5DS), in contrast to the whole genome shotgun data, where a number of best hits were previously mapped to one of the other sub-genomes (10-11% of hits to the A genome, and 6-8% to the B genome).

**Figure 8:**
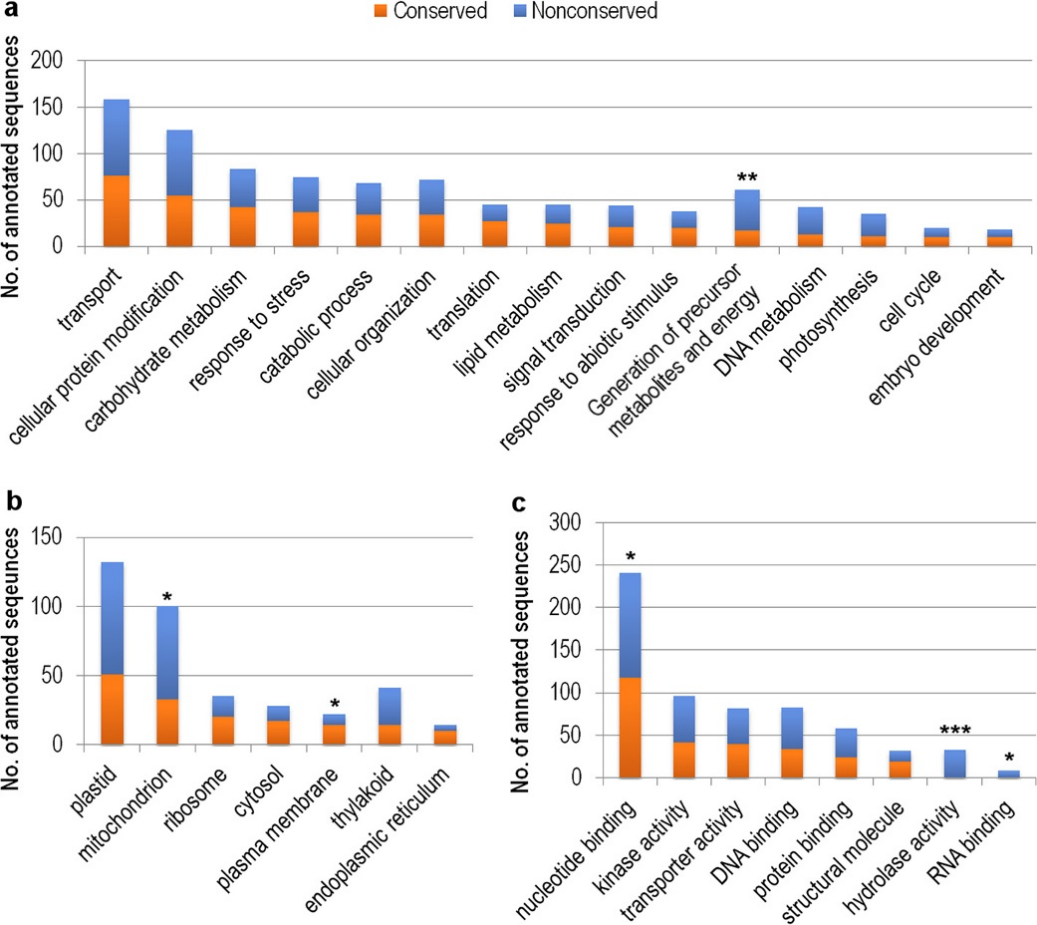
**Functional annotation of 5D gene models.** The total number of annotations for all Gene Ontology terms that matched 20 or more gene models is summarized in the **a**. Biological Process, **b**. Cellular Component, and **c**. Molecular Function categories. Significant differences between conserved and non-conserved gene model annotations for a given term are indicated by asterisks, deduced from Fisher’s exact test for two-tailed probabilities (*p-value <0.05, **p < 0.01, ***p < 0.001).

All the gene models built from both conserved orthologous genes and UniGene/UniProt sequences were also annotated for putative functions by assigning Gene Ontology (GO) terms from homologous sequences. The assignment of GO terms to each chromosome arm was largely similar, and, the most abundant terms found in each of the 3 ontologies are summarized in Figure 8. Some quantitative differences were observed between the terms assigned to conserved gene models and non-conserved gene-like sequences; for example, among Biological Process terms, ‘generation of precursor metabolites and energy’ was significantly enriched in non-conserved gene-like sequences (Figure 8a) and similar to the mitochondrion-related annotations among Cellular Component terms (Figure 8b), suggesting that chromosome 5D might have evolved genes with novel energy-related functions after the divergence of Triticeae tribe. Conversely, ‘plasma membrane’ annotations were more prominent among conserved gene models than non-conserved gene-like sequences. Finally, among Molecular Function terms, ‘nucleotide binding’, ‘hydrolase activity’ and ‘RNA binding’ were enriched in non-conserved gene-like sequences. In particular, ‘hydrolase activity’ annotations were exclusively derived from these non-conserved sequences at high statistical significance (Figure 8c, p-value = 1.01 × 10^-8^, Fisher’s exact test). The sequences of genes that have previously been cloned from chromosome 5D were also searched for in our dataset. As shown in Table 4, the important *Pina-D1* and *Pinb-D1* genes on 5DS, and *VrnD1* and *Lr1* on 5DL, were matched by several sequence reads covering 50-100% of the gene sequence and distributed along the length of the genes (Additional file 12), confirming their presence on these chromosome arms. In contrast only fragments were found of the *ADH1D* gene that has previously been mapped to 5DL [28] and the *VrnD3* sequence from *Ae. tauschii*, suggesting that these genes may not be actually present on *T. aestivum* 5D. *Lr1* gave a higher depth of coverage than expected (7× rather than 2-3×), indicating that there may be 2 or 3 genes with highly similar sequences on this chromosome arm. Many rRNA genes are present in multiple copies and likely to be masked as repeats; therefore, these gene sequences were also searched for in the unmasked reads. For the protein coding genes mentioned above no additional matching reads were found, but the *5S-RNA-D2* gene and the nucleolus organising region (*Nor-D3*) were detected at high depths of coverage.

**Table 4:** **Previously cloned genes identified in 5D survey sequence reads**

Gene	No. of matching reads	Total length	Matched length	Coverage (%)	Average depth
**5DS**					
***Pina-D1***	6	447	447	100.00	2.48
***Pinb-D1***	6	828	447	53.99	2.29
Nor-D3*	165	887	887	100.00	50.54
5S-RNA-D2*	71	486	486	100.00	32.59
**5DL**					
***Vrn-D1***	9	980	833	85.00	2.16
***ADH1D***	3	1140	235	20.61	1.60
***Vrn3***	1	1100	135	12.27	1.00
***Lr1***	90	4035	3958	98.09	7.00

*Repetitive reads were included in searches for these sequences.

## Discussion

A significant proportion of all human nutrition, either directly as a staple food or indirectly via use as animal feed, is provided by grasses of the Poaceae family, such as wheat, barley, oats, rye, rice, maize, sorghum, millet and sugarcane. Bread wheat (*T. aestivum*) is arguably the most important of these species, but has one of the largest and most complex genomes. With the increasing availability of next-generation sequencing technologies at reduced costs, a number of groups have reported sequence surveys of individual chromosomes, along with two genome-wide surveys, both by a whole-genome shotgun strategy [15] and, very recently, by Illumina sequencing of isolated chromosomes [16]. These surveys have great value for identifying putative protein-coding genes and, with the help of genetic mapping and synteny analysis, creating a virtual order of conserved genes along each chromosome, as first described in barley [29]. However, these studies acknowledge that the sequence data accumulated so far is of draft quality, which may contain errors and omissions to be resolved by subsequent studies.

As such, the data presented here provides an independent assessment of the structure and evolutionary features of chromosome 5D. Repetitive sequences, identified by a combination of sequence similarity and read depth comparisons, comprised 75.7% and 74.6% of the cumulative length of reads from 5DS and 5DL respectively (Figure 1 & Additional file 4). These values are within the range reported in similar surveys of other chromosome arms [10, 13] although a little lower than was observed in fully assembled BAC sequences from chromosome 3B (81.5%; [30]).

Putative tRNA genes predicted from unmasked and masked sequence reads from both arms revealed a striking abundance of tRNA^Lys^ species, followed by tRNA^Met^ and tRNA^Ser^ (Figure 2a,b); however, these abundances were largely attributed to an expansion driven by TE-capture and subsequent expansion of the repetitive elements. This abundance within repetitive elements may include many non-functional copies, but the putative tRNA gene distributions of the non-repetitive sequences could have functional implications. It has been proposed that a species-specific preference for the second position in a protein sequence exists for the majority of proteins starting with a Methionine (Met) residue, which may have an effect on the translation, and thus regulation, of the protein. This preference was shown to be more profound in plants, represented by *Arabidopsis thaliana*, which favoured Alanine (Ala) residues, followed by Serine (Ser) residues, at the second position [31]. Such a preference should also be reflected in tRNA gene abundances, which is supported by the tRNA^Met^ and tRNA^Ala^ predictions in this study. Additionally, wobble base-pairing arising from the degeneracy of the genetic code has recently been proposed as an intentional mechanism to temporally control the expression of proteins [32]. It is tempting to speculate that the abundance of certain tRNA genes may provide a more flexible use of anti-codons, and thereby wobble base-pairing as a means of gene regulation. Furthermore, the differential enrichment of chromosomes 5A and 5D for putative tRNA species is intriguing; among the polar, non-polar and charged amino acid classes, each chromosome appears to favor a different subset of tRNA species. While our current understanding of the wheat genome suggests transcriptional autonomy without the dominance of each sub-genome [16], the translational autonomy of individual chromosomes may present an interesting aspect of future research.

After eliminating the repetitive sequences, we found extensive homology to 1,493 genes from the fully sequenced grass genomes on 5DS and 2,829 on 5DL. We observed several differences in gene and synteny conservation between the two arms of the chromosome. In particular, the telomeric region of 5DS appears to have had a large accumulation of non-syntenic genes compared to the rest of the chromosome. A similar phenomenon was observed to differing degrees for chromosomes arms 1BS and 1AS [11] and proposed to be driven by TE activity. Furthermore, a higher proportion of conserved gene sequences on 5DL than 5DS had homology to 2 or more of the sequenced grass genomes (Table 3), and evidence for expression (Figure 1c-d). These observations might be explained by the observed introgression of non-syntenic gene sequences into the telomeric region of 5DS. If such sequences were introduced during TE replication, they are likely to be incomplete, and so accumulate more sequence mutations than functional genes. However, the impact of differing recombination rates or TE activity in other regions of the chromosome cannot be ruled out. Also intriguing is the observation that a higher proportion of gene sequences on 5DS than 5DL were conserved with both *B. distachyon & O. sativa* but not *S. bicolor,* while the opposite trend was observed for genes conserved with *B. distachyon & S. bicolor* only (Figure 1a-b). One possible explanation for this would be regional variations in the mutation rate of the chromosome 5D gene space at different stages of evolutionary history. The Panicoideae sub-family (sorghum) diverged from the Pooideae (*Brachypodium* & wheat) earlier than the Ehrhartoideae (rice) [33]. If the mutation rate of the 5DS gene space was greater between these two divergences, while that of the 5DL gene space was greater at some stage after the rice and wheat lineages diverged, it might give rise to the observed pattern. A more comprehensive study of such semi-conserved gene sequences would be valuable, to reveal whether such differences are widespread in the wheat genome. The locations of conserved genes on the other grass genomes enabled syntenic regions to be identified (Figures 3, 4) while mapping of known EST & SSR markers and comparison with syntenic regions from *Brachypodium* enabled the order of syntenic genes in the chromosome to be assessed (Figure 5). Using chromosome-specific sequences is particularly helpful in this case; by whole-genome shotgun sequencing, only one *B. distachyon* syntenic region could be mapped for 5D (on chromosome 1) due to the scarcity of genetic markers on the D genome [15], whereas, here, two different regions of chromosome 4 were also highlighted. The co-linearity of syntenic genes between 5DS and *B. distachyon* seems to have been well-maintained, with one possible inversion in the middle of the chromosome arm. In contrast, the peri-centromeric region of 5DL that is syntenic with Bd4 appears to have undergone two large-scale rearrangements. This is in contrast with 5AL, where the co-linearity with Bd4 is maintained but the region syntenic to Bd1 contains an inversion [10]. Comparison with *H. vulgare* 5H [9] further highlighted the conservation of large-scale co-linearity between Triticeae, but with many differences at the level of individual genes.

The reads matching conserved genes and UniGene/UniProt sequences were reassembled and mapped with *T. aestivum* transcriptome sequences, giving 982 high-confidence gene models on 5DS and 2,165 on 5DL, along with a smaller number of low-confidence gene models with more limited transcript coverage (332 and 810 respectively). In the IWGSC dataset, 598 high-confidence gene models were anchored to 5DS and 2,482 to 5DL, analogous to our conserved gene models. While the figures are in a similar range, the differences raise a question of interest to researchers planning to make use of these sequences: how reliable are the gene models? One way of answering this is to test their consistency between different datasets (Figure 7). While many gene models were highly consistent between studies, slight sequence differences were common, reflecting the biases of the sequencing technologies and assembly strategies used. In a genome where homoeologs may have up to 99% sequence identity [28] this can lead to conflicting chromosome location assignments. Comparing our 5D gene models with those from the IWGSC study, sequences of ≤98% nucleotide identity were just as likely to be found elsewhere in the genome as on 5D. Furthermore, even at 100% sequence identity, some gene models were assigned to other chromosomes. This comparison gives confirmation of the location of many genes on 5D, while highlighting some that require verification. Similarly, we were able to assign a number of gene models from the whole genome shotgun study [15] that were not previously mapped to a sub-genome to chromosome 5D. These issues illustrate the value of combining different sequencing strategies to give a more comprehensive survey of the chromosome, and highlight the need for a reference sequence assembly of the entire wheat genome, which has so far only been achieved for 3B [17].

Some of the putative ‘non-conserved gene-like sequences’ are likely to be pseudogenes or gene fragments, but they also include many expressed genes, as shown by the transcriptome mapping. These are of particular interest as the previous genome-wide sequence surveys focused on conservation with other grasses to define their gene models, and so may have omitted some of the genes that have diverged in protein sequence from their counterparts in other grass genomes and so have novel, Triticeae-specific functions. There is evidence for extensive intra-chromosomal gene duplications on 3B [17], and the enrichment for specific GO terms among these gene-related sequences on 5D (Figure 8) may suggest a similar recent expansion of specific gene families.

## Conclusions

In summary, the survey sequences presented here include 3,147 high-confidence and 1,142 low-confidence gene models for *T. aestivum* chromosome 5D including sequences orthologous with other grass genomes and others derived from more divergent sequences. Of the conserved orthologous genes, 2,138 were placed in a virtual gene order. These data are complementary to other *T. aestivum* sequence datasets, verifying some gene models, allowing some, for which no chromosome location was known, to be assigned to 5D, and highlighting others for which the current map location is questionable. Furthermore, evidence was found both for large-scale rearrangements of this chromosome compared to 5A and an accumulation of non-syntenic genes near the telomere of 5DS, highlighting that evolutionary processes have led to structural divergence between the wheat sub-genomes and even between different regions of the same chromosome. The detailed examination of 5D presented here suggests that there have been a large number of gene rearrangements and translocations since its divergence from *B. distachyon*, distributed throughout the chromosome but especially in the telomeric region of 5DS. These present opportunities for chromosome-specific marker development, and will be a valuable resource for the future mapping and reference-quality sequencing of this chromosome.

## Methods

### Isolation and amplification of wheat chromosome 5D

Liquid suspensions of intact mitotic chromosomes were prepared from a double ditelosomic line (2n = 40 + 2t5DS +2t5DL) of *Triticum aestivum* L. var. Chinese Spring according to [34]. The short and long arms of 5D were flow sorted and their purity checked by fluoresence *in situ* hybridization with probes for Afa family and telomeric repeats as previously described [4, 13]. Chromosomal DNA was purified and subsequently amplified by MDA using the illustra GenomiPhi DNA Amplification kit (GE Healthcare, Chalfont St. Giles, United Kingdom) according to [35].

### Next generation sequencing

Shotgun libraries were prepared from each chromosome arm using the GS FLX Titanium Rapid Library kit, quantified by enrichment titration and amplified and sequenced using GS FLX Titanium emPCR and Sequencing kits according to the manufacturer’s recommendations (all Roche 454 Life Sciences).

### Characterization of repetitive elements of 5D

The 454 sequence reads from each arm of chromosome 5D were searched for repeats using RepeatMasker version 3.3.0 [36]. A custom RepeatMasker database was produced by combining known Triticeae repeat sequences from TREP release 10 [37] with Repbase Update release 15.11 [38, 39] and TIGR Plant Repeat Databases [40, 41]. Comparison of repeat masked reads with those assembled into contigs was carried out using in-house Perl scripts.

### Gene, genetic marker and assembled transcript sequence resources

All predicted protein sequences for grass genome reference sequences were retrieved from the following sources: *B. distachyon* genome annotation v1.2 [33, 42], *O. sativa* genome assembly IRGSP-1.0 [43, 44] and *S. bicolor* genome assembly v1.4 [42, 45]. Cytogenetic map positions of EST and SSR markers found on 5D were retrieved from URGI [46], and EST sequences from wEST [47]. SSR sequences were kindly provided by P. Sourdille. EST assemblies for *T. aestivum, H. vulgare, P. virgatum, S. officinarum,* and *Zea mays* were downloaded from UniGene [48] while known protein sequences from the same species were obtained from the UniProt KnowledgeBase [49]. Model gene assemblies for *T. aestivum* whole genome shotgun sequences were downloaded from CerealsDB [50], and those for the IWGSC chromosome-specific gene models from [51].

### Sequence similarity searches

All sequence similarity searches were carried out using the BLAST+ command line applications, v.2.2.27 [52]. Non-repetitive 5D survey sequence reads were compared with *T. aestivum* mitochondrial and chloroplast genome sequences, and all reads with ≥95% identity to one of the organellar genomes over ≥75% of the read length were removed. The remaining reads were compared to protein sequences from other plant genomes and UniProt sequences using blastx with an e-value cutoff of 1e^-6^, and only hits of at least 75% amino acid similarity over a minimum length of 30 amino acids were retained. The search was also carried out in the reverse direction using tblastn with the same cutoffs; only matches that were the best hit for a given sequence read in both searches were retained. UniGene sequences were searched using blastn with an e-value cutoff of 1e^-30^, requiring ≥75% sequence identity over at least 90 nt, and only considering the best hit for each sequence read. For UniGenes from *T. aestivum,* the minimum sequence identity requirement was increased to 95%. Where 2 or more identical reads aligned to a protein or UniGene with the same start and finish points, these were considered to be amplification artifacts [11] and all but one copy of each sequence was eliminated using an in-house Perl script. Perl and Matlab scripts were also used to, collate the results of different BLAST searches for each sequence read, incorporate marker information, and assemble the genome zipper. EST, cloned gene and SSR sequences within 5D sequence reads were identified using blastn, with positives having at least 95% sequence identity over 30 or more nucleotides. Putative tRNA genes were predicted using tRNAscan-SE program [53] using the default parameters for eukaryotic genomes.

### Gene modelling

For modelling of the gene coding sequences, all non-repetitive reads were reassembled using gsMapper software (Roche 454 Life Sciences). A ‘reference genome’ was constructed from the coding sequences of the conserved genes in other genomes previously identified using blast searches in the following order of precedence: *B. distachyon,* rice, sorghum, UniGene. Sequence reads from 5D were mapped onto this reference with auto trimming on, overlap length and minimum contig length both set to 40 nt, and other parameters at default values. For a small number of reads that matched UniProt entries but no conserved genes or UniGenes, it was not possible to define a reference nucleotide sequence. These reads were instead extracted and used for *de novo* assembly with gsAssembler, using the same alignment paramaters. When multiple contigs mapped to different sections of the same gene/Unigene, the sequences were condensed using a Perl script into a single model, with gaps filled by strings of ‘n’.

Whole *T. aestivum* cv. Chinese Spring assembled transcriptome sequences also generated on the 454 GS FLX platform (NCBI BioProject Accession PRJEB3008; [15, 50]) were then used to screen the gene models by blastn, with a minimum 95% sequence identity. The Blast hits were collated using Perl scripts and the percentage of bases from each model covered by transcripts calculated. Models with 60-100% transcript coverage were classed as high-confidence, those with 20-60% as low-confidence, and the remainder were eliminated.

Gene models were compared to those from other wheat sequence datasets using blastn with a minimum 95% sequence identity and an e-value cutoff of 1e^-20^. When multiple best hits with the same e-value were obtained from homoeologous locations, the hit located on 5D was selected.

### Visualization and annotation

Heatmaps were drawn using Matlab, circle plots created using Circos software [54] and a virtual gene order was constructed using the ‘genome zipper’ approach [8]. Linear comparison of the gene order on different chromosomes was visualized using Strudel [55]. For Gene Ontology (GO) annotation, gene models were used as the query to search for functionally annotated sequences in the NCBI *Viridiplantae* non-redundant protein database with an e-value cutoff of 1e^-6^, and retaining the best hit for each query sequence, and using the output option –outfmt 5. The blast results table was imported into Blast2GO Software [56], where mapping and annotation was performed using default parameters, and the results streamlined for plants using GO-Slim. Fisher’s exact test (two-tailed) was used to calculate statistically significant differences in the number of annotations for each term between conserved gene models and non-conserved gene-related sequences, compared to the total number of other GO annotations in the same category (Biological Process, Cellular Component or Molecular Function).

### Availability of supporting data

The raw sequence data are available in the EBI European Nucleotide Archive, study no. ERP002330 [http://www.ebi.ac.uk/ena/data/view/ERP002330]. All other data sets supporting the results of this article are included within the article and its additional files.

## Electronic supplementary material

Additional file 1: **Flow cytometric isolation of 5D chromosome arms.** Histogram of fluorescence intensity (flow karyotype) obtained after flow cytometric analysis of DAPI-stained mitotic chromosomes isolated from double ditelosomic line 5D of wheat cv. Chinese Spring. (PPTX 247 KB)

Additional file 2: **Identifying 5D repeats by assembly depth and sequence similarity.** Methods used for identifying and eliminating repetitive sequences. (DOCX 20 KB)

Additional file 3: **Assembly of 5D survey sequences detects collapsed repeats.** Distribution of assembled reads by contig depth (calculated as contig length/total length of assembled reads). Both chromosome arms show a peak contig depth of 3–4, but give many contigs of much higher depth. **b**, **c.** Contigs of depth 5 or more contain an increased proportion of known repeat sequences in both 5DS (**b**.) and 5DL (**c**.) assemblies. (TIFF 188 KB)

Additional file 4: **Known wheat repeat families found in chromosome 5D.** Repeats were classified using RepeatMasker. Repeat content is calculated as the cumulative length of sequences masked by a given repeat family as a percentage of the total length of the sequence reads. (XLS 28 KB)

Additional file 5: **Genes sampled by chromosome 5D sequence reads.** Complete lists of all conserved grass gene models found on chromosome 5D (spreadsheets labelled 5DLconserved and 5DSconserved), and UniGene/UniProt sequences corresponding to putative lineage-specific genes (5DLnonconserved and 5Dsnonconserved). (XLS 1 MB)

Additional file 6: **Distribution of 5D gene orthologs on other grass genomes.** Heat map showing the distribution of 5D sequence reads with homology to genes on *B. distachyon* (Bd), *O. sativa* (Os) &*S. bicolor* (Sb) chromosomes. Heat map was drawn using a sliding window approach, with a window size of 500 kb and a step size of 50 kb. For the colour scale Min = 0 genes /100 kb, but the maximum is specific to each species as follows: Max genes /100 kb = 47.2 (Bd), 26.2 (Os), 34.25 (Sb). (TIFF 542 KB)

Additional file 7: **Chromosome 5D virtual gene order.** 5DSgenomezipper: Virtual gene order for chromosome arm 5DS, ordered based on co-linear regions of B. distachyon and deletion bin-mapped ESTs. 5DLgenomezipper: Virtual gene order for chromosome arm 5DL, ordered based on co-linear regions of B. distachyon and deletion bin-mapped ESTs. Region boundaries: summary of ranges of *B. distachyon* syntenic genes located on each chromosome arm, colour-coded according to each deletion bin. Grey indicates sections located on the boundary between two markers, that could not be definitively assigned to either deletion bin. (XLS 1 MB)

Additional file 8: **Coverages of two** ***Brachypodium*** **genes by non-syntenic 5D sequence reads.** Representative figures showing distribution of reads matching two non-syntenic genes, Bradi1g17710 (left), and Bradi1g32050 (right). Bradi1g17710 was evenly covered by 19 5DS reads, whereas Bradi1g32050 was covered by 9 5DL reads all of which are at the 5’ of the gene. Consequently, Bradi1g17710 was concluded to have an ortholog in 5DS, whereas the matches to Bradi1g32050 were concluded to be artefactual. On both diagrams x-axis shows the gene length in nucleotides. (TIFF 25 KB)

Additional file 9: **Non-syntenic 5D sequence reads.** Details of the evaluation of 5D sequence reads matching genes from the three model grass genomes that are found outside the 5D syntenic regions. (XLS 441 KB)

Additional file 10: **Gene model composition and statistics.** Assembly and transcript mapping details of the High- and Low-Confidence gene coding sequence models from both chromosome arms. (XLS 854 KB)

Additional file 11: **Gene model sequences.** Sequences of all gene coding sequence models in fasta format. (TXT 5 MB)

Additional file 12: **Coverage of genes previously cloned in 5D by 454 reads.** Details of 5D sequence reads matching cloned genes from this chromosome, and diagrams showing sequence coverage. Each coloured bar represents the gene segment covered by a single sequence read. (XLS 165 KB)

## Abbreviations

BAC: Bacterial Artificial Chromosome
BES: BAC-End Sequence
EST: Expressed Sequence Tag
GO: Gene Ontology
IWGSC: International Wheat Genome Sequencing Consortium
MDA: Multiple Displacement Amplification
SSR: Simple Sequence Repeat.
