# Supplementary material for: Next-generation sequencing of flow-sorted wheat chromosome 5D reveals lineage-specific translocations and widespread gene duplications
Source: BMC Genomics. 2014 Dec 9;15(1):1080. doi: 10.1186/1471-2164-15-1080 (PMC4298962; doi:10.1186/1471-2164-15-1080)
Supplement: Supplementary file 1 — Additional file 1: Flow cytometric isolation of 5D chromosome arms. Histogram of fluorescence intensity (flow karyotype) obtained after flow cytometric analysis of DAPI-stained mitotic chromosomes isolated from double ditelosomic line 5D of wheat cv. Chinese Spring. (PPTX 247 KB) [file 12864_2014_6895_MOESM1_ESM.pptx]

## Slide 1
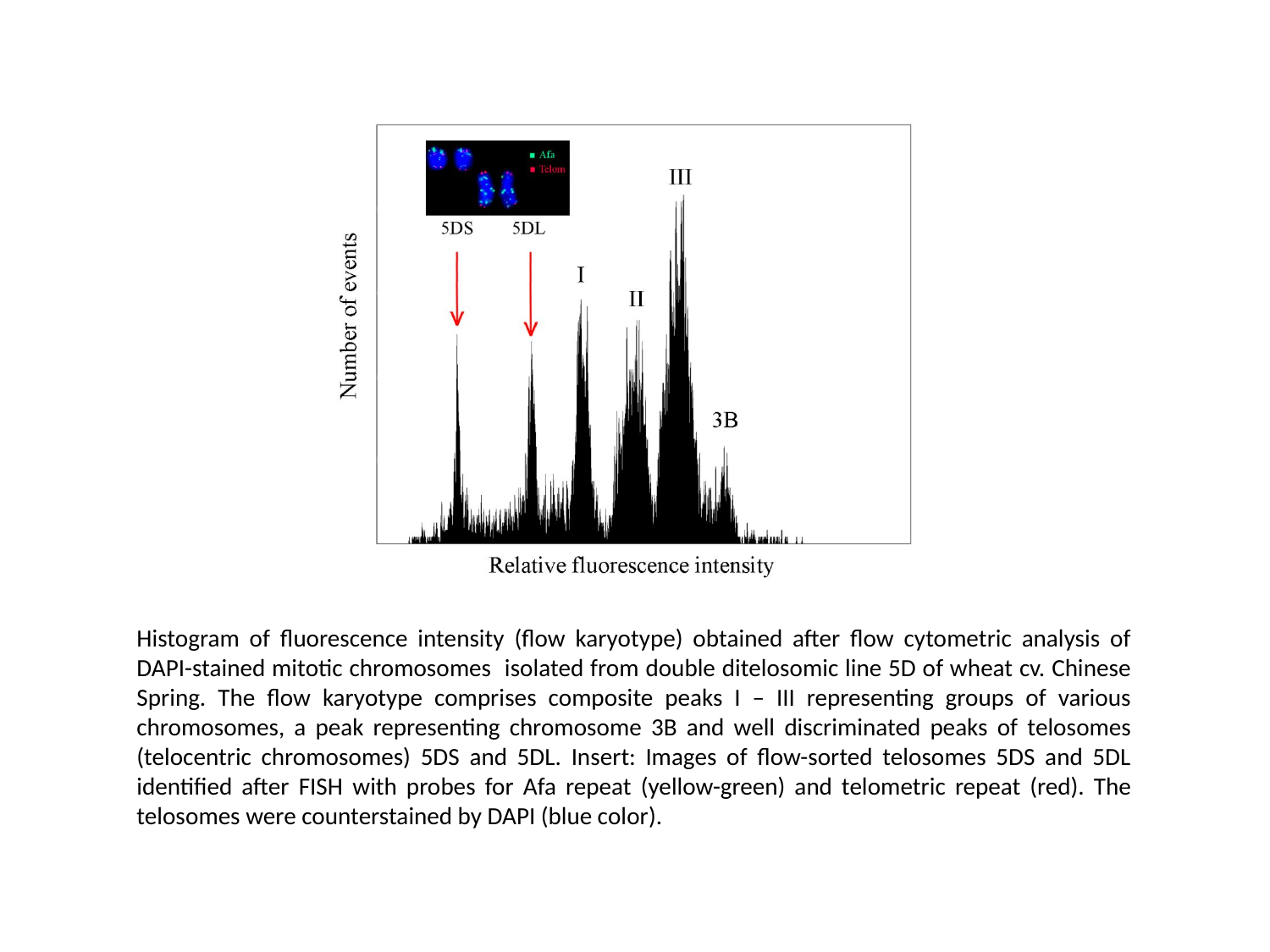

Histogram of fluorescence intensity (flow karyotype) obtained after flow cytometric analysis of DAPI-stained mitotic chromosomes isolated from double ditelosomic line 5D of wheat cv. Chinese Spring. The flow karyotype comprises composite peaks I – III representing groups of various chromosomes, a peak representing chromosome 3B and well discriminated peaks of telosomes (telocentric chromosomes) 5DS and 5DL. Insert: Images of flow-sorted telosomes 5DS and 5DL identified after FISH with probes for Afa repeat (yellow-green) and telometric repeat (red). The telosomes were counterstained by DAPI (blue color).
