# Supplementary material for: Next-generation sequencing of flow-sorted wheat chromosome 5D reveals lineage-specific translocations and widespread gene duplications
Source: BMC Genomics. 2014 Dec 9;15(1):1080. doi: 10.1186/1471-2164-15-1080 (PMC4298962; doi:10.1186/1471-2164-15-1080)
Supplement: Supplementary file 2 — Additional file 2: Identifying 5D repeats by assembly depth and sequence similarity. Methods used for identifying and eliminating repetitive sequences. (DOCX 20 KB) [file 12864_2014_6895_MOESM2_ESM.docx]

**Identifying 5D repeats by assembly depth & sequence similarity**

**Assembly distribution analysis**

Assuming that the reads at 1.34x and 1.61x coverage are distributed according to a Poisson distribution, 66.7% of 5DS and 68.4% of 5DL sequences are expected to be represented at least once in the dataset. This estimate was tested by carrying out similarity searches for the sequences of 518 Expressed Sequence Tag (EST) and Simple Sequence Repeat (SSR) markers that were previously deletion bin-mapped to chromosome 5D (Linkiewicz *et al.*, 2004; Sourdille *et al.*, 2004). Of these, 86/123 = 70% of the 5DS-specific and 270/392 = 69% of the 5DL-specific markers were detected in the corresponding sequence dataset. These values are close to the statistical estimates, suggesting that this dataset includes nearly 70% of the complete 5D chromosome sequence.

Assemblies of the sequence reads were carried out using gsAssembler software (Newbler 2.6, Roche 454 Life Sciences) with an empirically determined minimum overlap identity of 95%, and reads restricted to a single contig. Default values were used for other parameters.

More than half of the sequence reads were assembled, but the resulting contigs only covered 13.5% of the expected length of each chromosome arm (Table 2). It was observed that while the average depth of both assemblies was 2.2, many contigs had a much greater average depth (Additional File 3), suggesting that they consist of collapsed repeats.

**Sequence similarity analysis**

The sequence reads were analyzed using RepeatMasker, which identified ~70% of each chromosome arm as matching known repetitive elements (Additional File 4). The distribution of repeat families on each arm was similar, with Gypsy family retroelements accounting for over 46% of all the survey sequences, followed by large contributions (8-11% each) of Copia retroelements and CACTA transposons. The RepeatMasker output was used to assess the presence of known repetitive element sequences in contigs of different depths. As shown in Additional File 3 (b and c), contigs with low assembly depth contain <70% known repeat sequences; as contig depth increases from 3 to 6, the known repeat content increases to over 80%. Based on this observation and the average sequence coverage of 1.34-1.61x, all contigs with a depth of 5 or more were classified as collapsed repeats, with the unmasked sequences from these contigs being potential novel wheat repetitive elements. Sequence reads that completely assembled into these high depth contigs (21.18% of 5DS and 19.31% of 5DL reads) were excluded from subsequent analyses. For the remaining reads, known repeat sequences were masked with runs of N.

**References**

Linkiewicz AM, Qi LL, Gill BS, Ratnasiri A, Echalier B, Chao S, Lazo GR, Hummel DD, Anderson OD, Akhunov ED, Dvorák J, Pathan MS, Nguyen HT, Peng JH, Lapitan NL, Miftahudin, Gustafson JP, La Rota CM, Sorrells ME, Hossain KG, Kalavacharla V, Kianian SF, Sandhu D, Bondareva SN, Gill KS, Conley EJ, Anderson JA, Fenton RD, Close TJ, McGuire PE, Qualset CO, Dubcovsky J: **A 2500-locus bin map of wheat homoeologous group 5 provides insights on gene distribution and colinearity with rice.** *Genetics* 2004, **168:** 665–676.

Sourdille P, Singh S, Cadalen T, Brown-Guedira GL, Gay G, Qi L, Gill BS, Dufour P, Murigneux A, Bernard M: **Microsatellite-based deletion bin system for the establishment of genetic-physical map relationships in wheat (*Triticum aestivum* L.).** *Functional & Integrative Genomics* 2004, **4:** 12-25.
