# Supplementary figures and images for: Next-generation sequencing of flow-sorted wheat chromosome 5D reveals lineage-specific translocations and widespread gene duplications
Source: BMC Genomics. 2014 Dec 9;15(1):1080. doi: 10.1186/1471-2164-15-1080 (PMC4298962; doi:10.1186/1471-2164-15-1080)

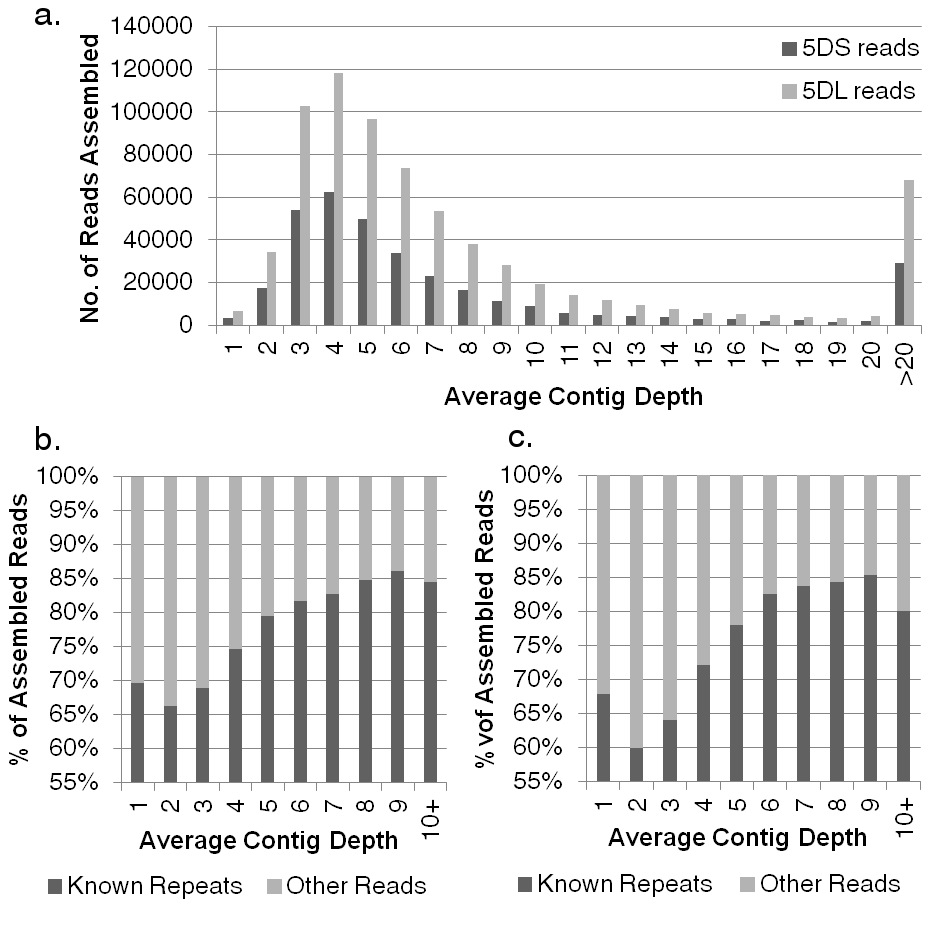

Supplement: Supplementary file 3 — Additional file 3: Assembly of 5D survey sequences detects collapsed repeats. Distribution of assembled reads by contig depth (calculated as contig length/total length of assembled reads). Both chromosome arms show a peak contig depth of 3–4, but give many contigs of much higher depth. b, c. Contigs of depth 5 or more contain an increased proportion of known repeat sequences in both 5DS (b.) and 5DL (c.) assemblies. (TIFF 188 KB) [file 12864_2014_6895_MOESM3_ESM.tiff]

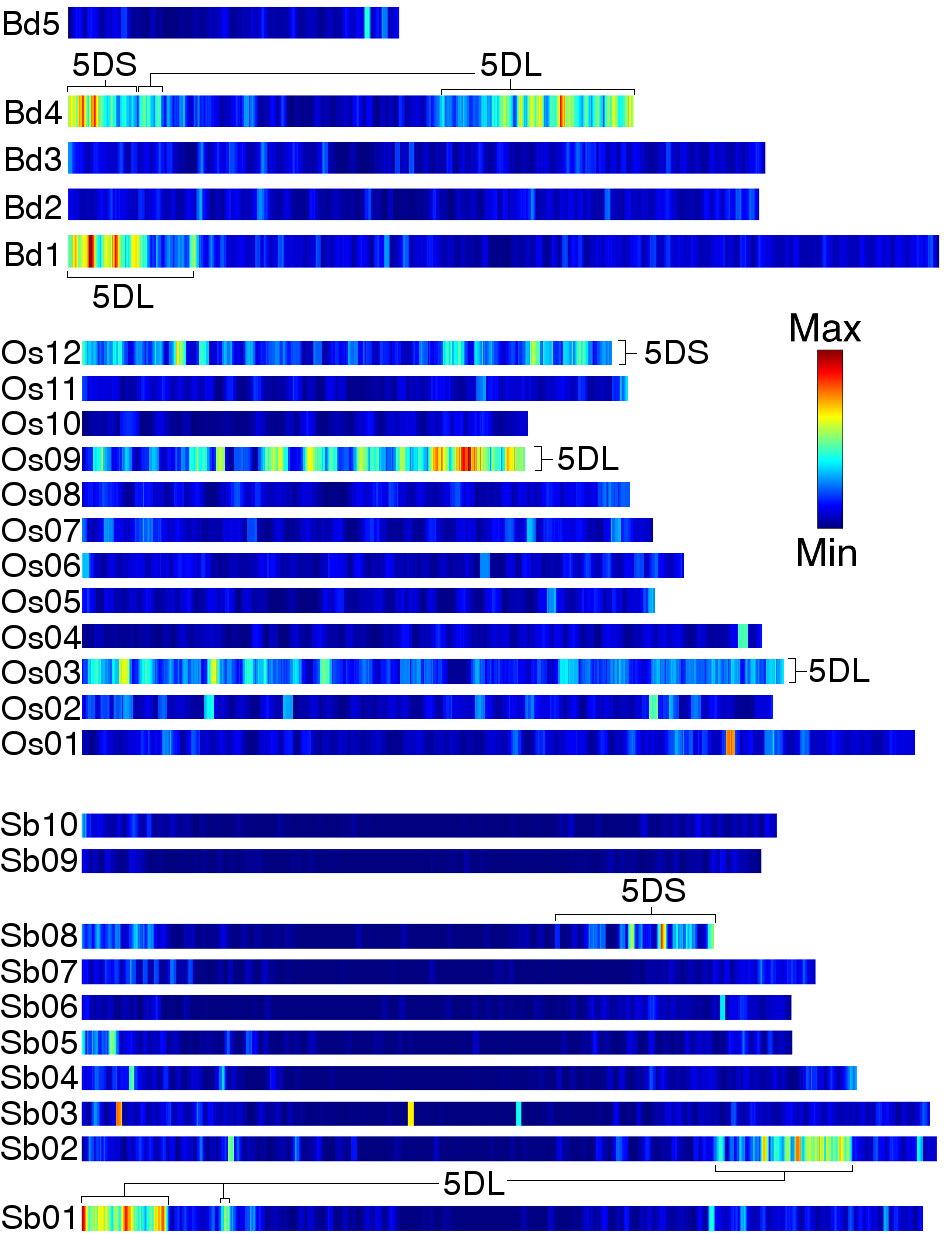

Supplement: Supplementary file 6 — Additional file 6: Distribution of 5D gene orthologs on other grass genomes. Heat map showing the distribution of 5D sequence reads with homology to genes on B. distachyon (Bd), O. sativa (Os) &S. bicolor (Sb) chromosomes. Heat map was drawn using a sliding window approach, with a window size of 500 kb and a step size of 50 kb. For the colour scale Min = 0 genes /100 kb, but the maximum is specific to each species as follows: Max genes /100 kb = 47.2 (Bd), 26.2 (Os), 34.25 (Sb). (TIFF 542 KB) [file 12864_2014_6895_MOESM6_ESM.tiff]

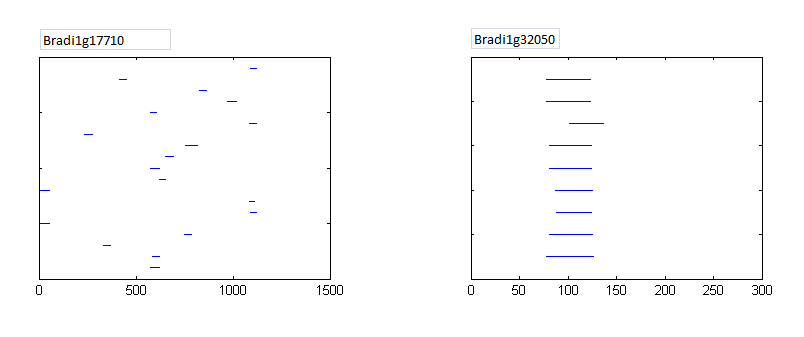

Supplement: Supplementary file 8 — Additional file 8: Coverages of two Brachypodium genes by non-syntenic 5D sequence reads. Representative figures showing distribution of reads matching two non-syntenic genes, Bradi1g17710 (left), and Bradi1g32050 (right). Bradi1g17710 was evenly covered by 19 5DS reads, whereas Bradi1g32050 was covered by 9 5DL reads all of which are at the 5’ of the gene. Consequently, Bradi1g17710 was concluded to have an ortholog in 5DS, whereas the matches to Bradi1g32050 were concluded to be artefactual. On both diagrams x-axis shows the gene length in nucleotides. (TIFF 25 KB) [file 12864_2014_6895_MOESM8_ESM.tiff]
